# Supplementary material for: Speeding-up while growing-up: Synchronous functional development of motor and non-motor processes across childhood and adolescence
Source: PLoS One. 2021 Sep 15;16(9):e0255892. doi: 10.1371/journal.pone.0255892 (PMC8443039; doi:10.1371/journal.pone.0255892)
Supplement: Attachment — Submitted filename: Response-to-reviewers.docx [file pone.0255892.s001.docx]

Please use the space provided to explain your answers to the questions above. You may also include additional comments for the author, including concerns about dual publication, research ethics, or publication ethics. (Please upload your review as an attachment if it exceeds 20,000 characters)

----------------------------------------

**Responses to Reviewers**

Reviewer #1: In this manuscript, the authors report the results of a study on the development of information processing speed during childhood and adolescence. A common Simon task was used, in which EMG was measured. Based on the EMG signal “premotor time” and “motor time” could be studied separately. The change in PMT and MT could be described by a negative exponential function, which indicated a similar development trajectory for both parts of the process. The researchers argue that this could indicate a common maturation factor. This idea is consistent with the global maturation theory, but contrasts with the finding that different brain regions mature at different rates. They also speculate that the common decay could be the result of a speed optimization mechanism whereby the faster processes are adapted to the speed of the slower processes.

This is an interesting and relevant work for researchers in developmental psychology. The article is well-written, the method seems solid (but see major comment below) and the results are clear.

I have only one general and a few smaller comments.

The study relies heavily on the assumption that PMT and MT can be separated on the basis of EMG.
1. Can the authors provide the necessary evidence for this? What is the validity of this method? What is premotor and motor here? These terms should be clearly operationalised.

**Response:** The reviewer is right that we went too quickly on this RT fractionation and what the two RT intervals could represent. We now review in more details the literature accumulated so far and what is known on the functional interpretation of these two intervals.

2. Related to this, in the discussion PMT and MT are linked to association cortices and motor brain regions, respectively. Since EMG measures polarization at the periphery, it is unclear to me to what extent you can make that link between EMG timing and brain processes.
If the authors can clarify this method / concept, this will facilitate interpretation of the results and discussion. If necessary, the limitations of this method or the associated assumptions can be acknowledge as a limitation at the end of the discussion.

**Response:** We now describe in more details what can be inferred on these two intervals, and we believe this clarifies the point raised by the reviewer. Importantly, although MT is measured at the periphery, it also reflects more central processes, and, as argued now, is sensitive to strategic control.

Minor points:
L49: Throughout the paper you refer to “cognitive function”. Whether you call reaction time and the underlying processes “cognitive” or “motor” may depend on your background. I would recommend adding a short footnote at the beginning of the paper to clarify what you mean by “cognitive”.

**Response:** As a footnote at page 3 we added a definition “Cognitive functions are broadly defined here as mental processes underlying perception, attention, learning, memory, reasoning, decision making, problem solving, action selection and control etc...”

L59, typos: a non-linear evolution characterizeS the maturation…., If so, WHETHER they have
L61-62: Please elaborate or provide a number of examples to clarify what you mean with tasks and tasks conditions here.

**Response:** In parenthesis (here in bold) we added examples of tasks used in previous studies “Previous attempts aiming at delimiting different processes aggregated in RT measure relied on comparing tasks or task conditions [1-2] associated to experimental manipulations **(for example: simple and choice RT tasks, visual search, mental rotation, same-different judgement tasks, Stoop task or classification task)** though to affect specific component processes.”

L64, typo: changes of RT WERE
L71: Please explain what you mean with “insertion of processes” and the “insertion hypothesis”

**Response:** Actually, the explanation was just before the definition of “insertion of processes”. As this was not clear, we moved the term a bit earlier in the sentence (changes are in bold).

“However, subtracting the RT of one condition from the other to draw inference on the assumed manipulated process (often termed “subtractive method”), requires additional assumptions. **First, one has to assume “pure insertion” of processes, that is** a mental process can be added or omitted from the processing chain without changing the duration of other processes [3]. In the developmental literature, it must further be assumed that 1) the nature of information processes engaged in a task are identical for all ages [4] and 2) the processes inserted are the same for all ages.”

L71, typo: do you mean “the quantity OR nature of information”?

**Response:** We removed the word “quantity” and “or” (see above):

L81: write LRP in full please
L106-107: This statement requires scientific evidence / support. See major comment.

**Response:** The reference has been added.

L142-143: The sample eventually consisted of 116 children, across a rather wide age range. This is considered to be small for behavioural research. I appreciate that the effects in your study are large, but I would ask to address the size of the sample briefly.

**Response:** For purely behavioral research, the sample studied would indeed be considered as rather small. In our recent study on children in which we applied uniquely behavioral measures (reaction times and errors) we have tested 360 children belonging to 10 age groups. Electromyography, however, is a much more demanding method than purely behavioral methods. It required substantial time investment (installations of electrodes, testing appropriate hand position, explication, and training with the subject how to behave to obtain a good signal) and also the experiment was conducted by two experimenters, one who explained and monitored task performance and the other one who monitored online EMG signal and asked the child to relax in case the signal was getting too noisy. Also, the initial sample of participants was larger (148 children) than the final sample (114 children). The fact that for few children abandoned the experiment or could deliver a good EMG signal shows that the procedure was demanding for children. Although it is indeed at the lower end of the acceptable number of participants per group, given the constraints, it is a good compromise between experimental load and power.

L190: Is my understanding correct that the time between the appearance of the fixation point and the stimulus was fixed? I can imagine that such a fixed period of time can give rise to anticipation. Have you checked for this? How did you deal with this?

**Response:** The interval between the fixation point and the stimulus onset was fixed. However, the side of stimulus presentation, its color as well as the side of required response was random. Therefore, subject could anticipate neither the nature of the stimulus nor the correct response.

L241: I admit that I am not a specialist in this matter, but in my opinion the Data Analysis section is clear and very well written.

**Response:** Thank you very much. We put lot of effort to make the data analysis description as clear as possible.

L266: Is it possible to include this in the OSF page?

**Response:** Yes, the document published on OSF page includes now also the analysis with parameter "a" free to vary.

L295, typo ?: While fitting eq 1 to THE data provideS essential information ..
L301, typo:… all variableS…
L321, typo: ... to which PMT correspondS ...
L367, Table 2, typo: First parameter b in lowercase
L439: bayes factor previously written as Bayesian Factor. Remain consistent.
L486: Clarify what you mean with tasks comparisons or provide an example (see also L61-62)

**Response:** Prompted by this comment we modified the following sentence in the introduction:

“Previous attempts aiming at delimiting different processes aggregated in RT measure relied on comparing tasks or task conditions [1-2] associated to experimental manipulations **(for example: simple and choice RT tasks, visual search, mental rotation, same-different judgement tasks, Stoop task or classification task)** thought to affect specific component processes.”

L519: Efficiency of various RT processes and brain maturity. At first, I couldn't fully follow, but later it became clear to me that this referred to brain function vs. brain structure. It seems useful to me to explain this briefly.

**Response:** In order to clarify this issue, at the beginning of the paragraph about functional and physiological maturity, we added the following sentence:

**“**The maturity of the brain is most often studied at two levels independently. One level is related to the functional maturity, that is to the efficiency of cognitive processes at fulfilling their function. The second level is related to physiological maturation, that is to the structure of the brain in terms of white and grey matter characteristics.”

L544: Within or before your concluding paragraph, a short section with the limitations of your work (e.g. assumptions related to the separation of PMT / MT based on EMG, sample size) is required. Here, it is important to note that your conclusions related to development are inferred from cross-sectional data. In that context it should be noted that you talk about biological processes (structural maturation and functional efficiency) while you have classified the groups based on chronological age. We know that there is great individual variation in timing and rate of maturation. These are minor points, which need to be acknowledged nonetheless.

**Response:** Prompted by this comment, we inserted the following paragraph about limitations (at the end of discussion) :

« Pointing to possible limitations of the current study, its cross-sectional nature may conceal more subtle age-related effects in the maturation of the duration of RTs components. This must be considered especially as the final size of the age groups is rather modest and only chronological age was considered. Thus, important individual variations in processes maturation was not taken into account. Finally, the method of separating PMT and MT is ubiquitous, but the functional interpretation of these RT components is not. Whereas MT can be considered as reliably estimating motor component, the influence of motor processes on PMT cannot be ruled out. »

Thank you for your careful reading.

--------------------------------------------------------------------

Reviewer #2: The paper “Speeding-up while growing up: synchronous functional development of motor and non-motor processes across childhood and adolescence" examines changes in reaction time on a sample of children 6-14 years old. Using EMG, they fractionate the RT into pre-motor and motor components and show that the changes in both components develop at the same rate.

Overall, the paper is well done and I believe it makes a useful contribution to the literature. I have a couple of major comments and some other minor comments for improvement.

Major comments:
1. In the introduction, it would be helpful to explain what the theoretical bases of the premotor vs. motor components of RT are from prior work. For example, several studies show that many changes in RT are almost fully correlated with the premotor component, and very little with the motor component. Are there studies that show changes in the motor component of RT and if so, what would be the basis for such changes?

**Response:** The reviewer is right that this part needed clarification. We now review the currently known literature on EMG-based fractionation and what can be inferred from the two intervals. Notably, we report the effect of some experimental manipulations, classically considered as “cognitive” on MT, and argue that MT duration is under strategic control. We hope this addresses the point raised by the reviewer.

2. Given the research question, the averaging of all participants within an age-group throws away a lot of information. In addition, this also reduces the number of data points that need to be fit by the exponential, which explains the high R^2 values. The authors should provide more individual level dot plots (rather than just the group averages) and also report the robustness of this curve fitting to removing some of the data points (to avoid any effects of a few outliers).

**Response:** Prompted by this comment we repeated the fit of the exponential function on individual data points (see figure below). This analysis is now available on the OSF page (<https://osf.io/93rmd/?view_only=f0077de9ac33419cbe663cff6e4d413f>.). The reviewer is right that fitting the exponential function on individual data points instead of on mean across age groups would result in lower R^2 values, as they range around 0.81 for RT and PMT and around 0.60 for MT. However, when calculating the AIC values for the model in which the decay rate for all variables (RT, PMT, MT compatible and incompatible) was free to vary and the model in which all data points were fitted with the same decay parameter *c* (the *full* versus the *restricted* model), the *restricted* model (AIC = -4587.7) outperformed the *full* model (-4076.1).

We also included the footnote in the manuscript that mention this additional analysis (in the part *Assessing the exponential decay rate of RT, PMT, and MT across development*). However, in the manuscript we would like to keep the analysis on mean values for age groups, because keeping means per age group allows us to fix the parameters *a* of the exponential function (the asymptote), which yields the fitting function more restriction and ensures more reliable results. Also we think that for sake of clarity, results on the mean data point are easier to inspect.

Figure. The fit of the exponential function on individual data points. Gray solid lines represent the full model (the parameter c free to vary across all variables) and the black dashed lines represent the restricted model (data sets of all variables fitted with the same parameter *c*)

3. The last part of the Discussion about “slowing down” processes to ensure “optimal flow of information” seems to be extremely speculative.

**Response:** We agree with the reviewer that the proposed interpretation is speculative, but the results are, by themselves, intriguing. This proposition may open new perspectives and research hypothesis in the field. However, in agreement with the reviewer’s comment, we now clearly state that this proposition is speculative.

Are there any known mechanisms that could affect the motor component of RT in a task-specific manner?

**Response:** We now review in more detail what PMT and MT correspond to in the introduction, and which factors affect these two intervals. More specifically, we clearly indicate that even MT can be modulated by strategic control.

The authors should also consider if there are other simpler alternatives for changes in the motor component of RT (e.g., the vigor with which participants respond, which can potentially shorten the time between the EMG burst and the response).

**Response:** The notion of vigor in responding is an interesting concept. It is, however, more appropriate for movements. In the present study, response are isometric muscular activations, without actual movement, in which the notion of vigor is less obvious. Besides, and more importantly, describing the results in terms of vigor does not solve the key issue: why would vigor develop at exactly the same rate as premotor components? We are still lacking a functional interpretation of this co-development.

Also, including this extremely speculative part in the abstract also makes it sound like a “likely” explanation when there is very little evidence for this (at least at the moment).

**Response:** The abstract has been updated to downplay the importance of this speculative interpretation.

Minor comments:
1. Ln 99 – there seems to be a missing reference

**Response:** The reference has been added.

2. Ln 226 – the EMG onset detection algorithm could be explained in a bit more detail here so that the manuscript is more self-contained

**Response:** In the method section, we added a short description of the algorithm:

“In a nutshell, after pre-processing to increase the signal/noise ratio (through the computation of the Teager-Kayser energy), to detect the presence of an EMG burst, the signal is compared to baselines values: when it exceeds the mean plus n times the standard deviation (n is a free parameter adjusted for each participant), an EMG burst is considered to have occurred. Its onset and offset are then estimated through its “integrated profile”: the cumulative sum of the signal is calculated from which the cumulative sum of the uniform distribution (i.e. a straight line) is suppressed. The onset is localised at the minimum of this difference and the offset at the maximum.”

3. At several points in the manuscript, the authors refer to information that is available upon request – it might be easier to add this information as supplementary material (especially given that some of the material is already on OSF)

**Response:** Prompted also by reviewer 1 comment, we extended the document published on OSF page by the additional analyses we made (the exponential fits with asymptote free to vary). As to EMG detection algorithm, we described it in the paper together with the link address to download the algorithms.

4. Ln 248 – insensitive (instead of unsensitive)
5. The description of the Brinley plots could be motivated earlier (presenting the rationale for the analysis in the results section makes it much more difficult to anticipate for the reader)

**Response:** We inserted a passage about Brinley plots in the introduction:

“In addition, the performance of children in each age group will be compared to that of adults to calculate the extent to which children are slower than adults. This analysis, called a Brinley plot [27], was traditionally performed on average RT data under various experimental conditions and involved plotting the mean RT of children against that of adults. Here, within-task Brinley plots will be calculated by plotting the average RT, PMT, and MT of children against those of adults. The common exponential decay rate for RT, PMT, and MT should result in a linear relationship between these variables and provide additional support for the hypothesis about the same developmental rate of RT components.”

Thank you for revising this manuscript.

-----------------------------------------------------------------------------------------------------------------------

Reviewer #3: In this paper, the authors decomposed response time into reaction time (PMT: time interval between stimulus onset to EMG onset) and movement time (MT: time interval between EMG onset and behavioral response) in a modified Simon task and found that both PMT and MT follow the same developmental trajectory from 6 years of age to adulthood. This paper is well-written, methods are clearly described, and analyses are appropriate. I am sure that the results themselves will contribute to the literature of developmental science. However, I have a major concern regarding the conclusion inferred from the data and I strongly suggest the authors performing one additional piece of analysis to address this concern. I describe it below:

I believe that the major argument the authors attempted to make is that behavioral signatures of cognitive (PMT) and motor processes (MT) follow the same developmental trajectory, contradicting the fact that the physiological development landscapes between cognitive and motor related brain areas are distinct. MT largely reflects a motor process, my concern, however, is that PMT does not reflect non-motor processes only. Clearly, besides perception (which is actually trivial in compatible trials as perception in these cases is primarily driven by automatic processing), premotor processes include action selection, and movement preparation, etc., which are generally thought of as motor processing, and related to premotor cortex (see Wong et al. 2015 Motor planning). However, the neural evidence of non-motor process (i.e., cognitive control) in the discussion section is mostly about temporal and frontal cortex. Thus, the behavioral measurements demonstrated in this paper and the existing evidence of neural development are not an apples-to-apples comparison. Some experiments where PMT is dominated by cognitive processes could provide a better comparison. One candidate is go/nogo task or stop-signal task that requires only one response. In these cases, the pre-motor interval is largely about cognitive control and may not be contaminated by motor-related process such as action selection. However, I do NOT suggest any additional experiments. The other way to address my concern is to perform an additional piece of analysis on the existing dataset. Unlike PMT in compatible trials, PMT in incompatible trials consists of an additional cognitive process (i.e., response inhibition which is related to frontal cortex) that addresses the competition between automatic processing induced by the spatial location of stimulus and the computational processing induced by the identity of the stimulus. This cognitive control function could be inferred by the PMT difference between these two types of trials. The result that this PMT difference follows the same nonlinear developmental trajectory with MT would strongly support the authors’ argument. Otherwise, the current conclusion would be undermined.

**Response:** We performed this additional analysis of comparing the difference between PMT compatible and PMT incompatible with all other variables such as RT, PMT and MT in compatible and incompatible task conditions. First, we fitted the negative exponential function to this new variable (the difference between PMT incompatible and compatible (see the figure below). We then calculated the AIC values of model in which decay parameter *c* varied independently for all 7 variables and AIC of the model in which all 7 data sets were fitted with the same decay parameter *c* that was previously obtained when fitting 6 data sets (RT, PMT and MT compatible and incompatible). What we found was again, the model with the same decay rate of exponential function for all 7 data points had smaller AIC value (-594.51) than the model with the decay parameter c free to vary across all 7 data sets (-577.32). Thus, we concluded that better fit was obtained with the model assuming the same decay rate for all data sets and thus, the decrease of the exponential function is the same for all variables. This analysis can be found on the OSF page (part 4 entitled “Assessing models with 7 data sets: RT, PMT and MT compatible and incompatible (6x) and the difference between PMT compatible and PMT incompatible (1x)” in the jupyter notebook: : <https://osf.io/93rmd/?view_only=f0077de9ac33419cbe663cff6e4d413f>.).

We inserted the footnote in the manuscript mentioning this additional analysis (in the part *Assessing the exponential decay rate of RT, PMT, and MT across development*). However, we refrain from reporting this analysis in the body of the manuscript. This is because this analysis relies on a very strong assumption, that some processes can be inserted (or removed) without affecting the surrounding ones and in the present situation, that PMT incompatible takes longer than PMT compatible only because of an additional process of inhibition that can be extracted through the subtraction of the two conditions. Based on a large literature, we criticize this assumption, commonly made in cognitive and developmental psychology research, in the introduction. We hence cannot decently report such an analysis, even if it supports our view, in the manuscript.

Figure. The fit of the exponential function to the values of the difference between PMT incompatible and compatible task conditions. The colors of data points represent different age groups, from the 6 years old (darkest color) to young adults (yellow color).

Another comment:
I found the abstract is a bit misleading as half of it is talking about neural development, but there is no neural data at all in this paper. I would suggest to focus on the data and results instead but I will leave the decision to the authors.

**Response:** Prompted by this comment we have re-written the abstract. The new part is in bold:

« Describing the maturation of information processing in children is fundamental for developmental science. Although non-linear changes in reaction times have been well-documented, direct measurement of the development of the different processing components is lacking. In this study, electromyography was used to quantify the maturation of premotor and motor processes on a sample of 114 children (6-14 years-old) and 15 adults. **Using a model-based approach, we show that the development of these two components is well-described by an exponential decrease in duration, with the decay rate being equal for the two components. These findings provide the first unbiased evidence in favour of the common developmental rate of nonmotor and motor processes by directly confronting rates of development of different processing components within the same task. This common developmental rate contrasts with the differential physical maturation of region-specific cerebral gray and white matter. Tentative paths of interpretation are proposed in the discussion.** »

Thank you for your work on this revision.
