# Supplementary material for: Speeding-up while growing-up: Synchronous functional development of motor and non-motor processes across childhood and adolescence
Source: PLoS One. 2021 Sep 15;16(9):e0255892. doi: 10.1371/journal.pone.0255892 (PMC8443039; doi:10.1371/journal.pone.0255892)
Supplement: Attachment — Submitted filename: Response-to-reviewers.docx [file pone.0255892.s002.docx]

**Responses to Reviewers**

Reviewer #1: All my comments have been addressed. I only spotted a few typos:
L55: of A series of
L64: StRoop task

Response: Done, thank you for careful reading.

Reviewer #3: Thanks for your effort to address my previous comments. There are two suggestions to your analyses:

1. The error data (in percentage) seems not follow the normal distribution assumption for ANOVA (I assume that most participants have very low error rates and they are bounded between 0 and 1). You may at least provide the test for the normal distribution assumption or use some alternatives such Poisson or Beta regression depending on the distributions the data follow.

Response: In response to this comment, we now present in paper ANOVA on arsine transformed error rates. ANOVA performed on these new values confirmed the previous analysis, therefore in method section we just added that analysis was performed on arsine transformed error rates and we adjusted F and p values in the result section.

2. Since two exponential models used to fit the data are nested, the likelihood ratio test would give a formal statistical comparison between models, rather than an arbitrary comparison given by AIC. That is, you could statistically reject the hypothesis that the full model is better than the restricted one.

I believe that these two suggestions will not affect the significance of your study, but it would improve the validity of the statistical analyses.

Response:

We thanks the reviewers for pointing out this possibility. We have indeed added the likelihood ration test, which clearly confirms that the full model does not add any significant information to the fit. To get an idea of the weight of evidence in favor of the two model, we also computed the weighted AIC (see Wagenmakers & Farnell, 2004) which revealed that the restricted model is 82 more likely than the full one, given the data.
